# Estrogen, the Peripheral Immune System and Major Depression – A Reproductive Lifespan Perspective

**DOI:** 10.3389/fnbeh.2022.850623

**Published:** 2022-04-15

**Authors:** Elizabeth B. Engler-Chiurazzi, Wesley H. Chastain, Kailen K. Citron, Lillian E. Lambert, Divya N. Kikkeri, Sharhana S. Shrestha

**Affiliations:** ^1^Department of Neurosurgery, Clinical Neuroscience Research Center, Tulane Brain Institute, Tulane University School of Medicine, New Orleans, LA, United States; ^2^Department of Neurology, Tulane University School of Medicine, New Orleans, LA, United States

**Keywords:** estrogen, sex differences, major depressive disorder, peripheral immune system, mood

## Abstract

Major depression is a significant medical issue impacting millions of individuals worldwide. Identifying factors contributing to its manifestation has been a subject of intense investigation for decades and several targets have emerged including sex hormones and the immune system. Indeed, an extensive body of literature has demonstrated that sex hormones play a critical role in modulating brain function and impacting mental health, especially among female organisms. Emerging findings also indicate an inflammatory etiology of major depression, revealing new opportunities to supplement, or even supersede, currently available pharmacological interventions in some patient populations. Given the established sex differences in immunity and the profound impact of fluctuations of sex hormone levels on the immune system within the female, interrogating how the endocrine, nervous, and immune systems converge to impact women’s mental health is warranted. Here, we review the impacts of endogenous estrogens as well as exogenously administered estrogen-containing therapies on affect and immunity and discuss these observations in the context of distinct reproductive milestones across the female lifespan. A theoretical framework and important considerations for additional study in regards to mental health and major depression are provided.

## Introduction

Mood disorders, including major depressive disorder (MDD), are a significant global health issue (Krishnan and Nestler, 2008). Worldwide lifetime prevalence of mood disorders has been reported to be nearly 10% (Steel et al., 2014), and in 2010, the nearly 300 million global cases of MDD accounted for 8.2% of all disease-induced years lived with disability (Ferrari et al., 2013a). In the US alone, one in six adults will receive an MDD diagnosis in their lifetime and more than 13 million Americans experience a major depressive episode with severe impairment each year (Kessler et al., 2005; Ferrari et al., 2013b; Brody et al., 2018). Annual costs associated with this condition are estimated at ∼$210 billion (Greenberg et al., 2015). MDD is generally considered a brain-targeted disease associated with persistent sadness, guilt, anhedonia (reduced interest in rewarding stimuli), despair, and in some cases, suicide (Krishnan and Nestler, 2008). Due to a happenstance discovery of psychiatric patients showing improved mood when treated with monoamine oxidase inhibitors (Schildkraut, 1965), MDD has historically been associated with deficiencies in serotonergic (5-HT), dopaminergic, and noradrenergic signaling within limbic, reward, and brainstem structures (Krishnan and Nestler, 2008). Problematically, available pharmacologic treatments targeting these presumed dysregulated monoamine systems are associated with delayed and inadequate symptom alleviation in a large proportion of patients (Trivedi et al., 2006; Al-Harbi, 2012; Akil et al., 2018). This led the field to conclude that the pathology of MDD is more complex than previously appreciated, that the neurotransmitters thought to underlie MDD-associated brain pathology may not be the sole contributors to its presentation, *and* that the therapeutic interventions targeting these systems will likely remain insufficient at imparting symptomatic relief.

Emerging data strongly implicate additional mechanisms in the manifestation of mood disorders. As a result, many researchers have begun considering biological factors that could significantly contribute to the development and persistence of MDD. One of these factors is that of genetic sex and the accompanying differences in sex hormone secretion across the lifespan. A substantial amount of research attention has been paid to the role of sex hormones, especially the steroid hormone estrogen, in driving development of MDD in women (Wharton et al., 2012; Eid et al., 2019). In addition to sex hormones, converging data amassed over the past few decades also support significant immune contributions to brain function and mood (Leonard, 2010; Dantzer, 2018). Indeed, it is now accepted that inflammatory cascades mediated by innate and adaptive arms of the immune system significantly contribute to MDD, at least in some patient subsets (Maes, 2011; Wohleb et al., 2016; Herkenham and Kigar, 2017). Sex differences in the susceptibility to certain infections, the presence of sex hormone receptors on immune cells, and shifts in the function of the immune system during distinct periods of the reproductive lifespan all point to a critical role of sex hormones in modulating immunity (Pennell et al., 2012; Klein and Flanagan, 2016).

Given the known sex differences in the prevalence of mood disorders, emerging support for the immune system’s role in mediating susceptibility or resilience to psychosocial stress, and the potentially profound impacts of sex hormones (especially estrogens) on impacting immunity, the consideration of neuro-immuno-endocrine interactions in the context of mood and MDD across the female lifespan, is warranted. Here, we will review evidence regarding the mood impacts of these factors individually, describe shifts in immune responses during key reproductive milestones, highlight a few examples of potential autoimmune consequences of estrogenic stimulation in females, and summarize the small but growing collection of findings exploring the convergence of sex, sex hormones and immune function in the context of mood and MDD. Finally, we present important experimental considerations when the convergence of these factors is investigated.

## Manifestation of Disordered Mood Across the Female Lifespan: Role for Estrogens

Women shoulder a disproportionate burden of mood disorders and the role of estrogen in modulating mood has been well studied. Estrogens are generally thought to improve mood in many, but not all, circumstances. Below, we highlight major observations driving this conclusion. Though a thorough discussion of this extensive literature is beyond the scope of the current review, the reader is directed to several excellent reviews specifically addressing this topic (Wharton et al., 2012; Altemus et al., 2014; Eid et al., 2019; LeGates et al., 2019).

### Sex Differences in Depression

Differences in the prevalence of MDD, phenotypic manifestations of depression, and the efficacy of antidepressant therapy between the sexes are well established (Altemus et al., 2014; LeGates et al., 2019). Rates of MDD are substantially higher among females compared to males (Weissman and Klerman, 1977), though this sex difference appears to be critically dependent on age. Prior to puberty, boys are more likely to have a mood disorder than girls (Faravelli et al., 2013). This incidence shifts during the pubertal transition as girls display depression at a rate double that of boys between the ages of 15 to 19 (Faravelli et al., 2013). MDD is nearly twice as prevalent in adult women than men, at rates of 10.4 and 5.5%, respectively (Brody et al., 2018). However, following reproductive senescence during the fifth decade of life, aging men and women tend to have similar prevalence rates of mood disorders (Faravelli et al., 2013).

Throughout life, men and women may also differ in their MDD endophenotypes. Results of several studies, including the large-scale Sequenced Treatment Alternatives to Relieve Depression (STAR*D) trial, indicate that women display higher rates of atypical and anxious depressive phenotypes. These are characterized by increased appetite, weight gain, comorbid eating disorder, rumination, hypersomnia, gastrointestinal complaints, and a higher rate of past suicide attempts relative to male patients (Marcus et al., 2008; Shors et al., 2017). Men are more likely to display comorbid substance use coping strategies and have higher rates of successful suicide, likely due to their use of more lethal means (e.g., firearms). Reports of irritability and the melancholic depressive subtype are similar in both men and women (Marcus et al., 2008).

Finally, though MDD treatments are available, barriers to treatment access as well as intervention type playing a role in the realization of symptom relief (LeGates et al., 2019) leaves the affective symptoms of many patients poorly controlled. Indeed, a recent study assessing nearly 250,000 depressed adults noted that only about 30% of MDD patients obtained pharmacological antidepressant treatment within three months of diagnosis (Waitzfelder et al., 2018), and of those, antidepressant efficacy is often delayed and highly variable (Trivedi et al., 2006; Al-Harbi, 2012; Akil et al., 2018). Sex may account for some of this variability as women appear to experience better symptom remission from selective serotonin (SSRI) or norepinephrine reuptake inhibitors, while men respond better when treated with tricyclic antidepressants (LeGates et al., 2019). Sex differences were not readily observed among adult patients with refractory bipolar/MDD undergoing repetitive transcranial magnetic stimulation (Huang et al., 2008). This observation appears to be age- and hormone status-dependent. Older women may display a poor response to rTMS (Huang et al., 2008) or the SSRI, venlafaxine (Thase et al., 2005), and this effect was reversed by estrogen supplementation. Sex differences in response to newly developed, fast-acting, glutamatergic-modulating, antidepressant interventions such as ketamine, are only beginning to be assessed. Emerging findings suggests conflicting results. Some groups have reported needing lower ketamine doses in female rats to impact affective behaviors under basal conditions while others report that male mice may be more responsive than females following exposure to stress (Saland et al., 2017; Okine et al., 2020). This is noteworthy given the recent Federal Drug Administration approval of nasally-administered esketamine for MDD patients treatment-resistant to traditional antidepressant interventions FDA (2019). It is also important to note that these sex-specific antidepressant treatment responses could also be explained, at least in part, by differences in the observed MDD endophenotypes in men versus women described above; further interrogation of this possibility is needed.

### Hormone Effects on Depression and Mood During and After the Reproductive Years in Women

These observations along with the dynamic shifts in reproductive capacity that take place across the female lifespan implicate ovarian hormones in modulating mood and mood disorders in women. Indeed, between 10 and 80% of women experience mood disruptions that are related to their menstrual cycle, (Baker and Driver, 2007), and 3-8% of women can experience premenstrual dysphoric disorder, characterized by extreme premenstrual anxiety, decreased mood, and irritability (Robakis et al., 2019). These observations have been reported for the past several decades in both human and preclinical populations, though not all studies have consistently found an association between cycle stage and affect (Moos et al., 1969; Laessle et al., 1990; Jenkins et al., 2001; D’Souza and Sadananda, 2017; Sundström-Poromaa, 2018; Zhao et al., 2021). As well, the peripartum period is associated with dynamic shifts in sex hormone levels, and one of the most common complications of pregnancy, observed to impact one in seven mothers, are postpartum mood and anxiety disorders (Wenzel, 2016; Luca et al., 2019). Among menopausal women, (Maartens et al., 2002; Bekku et al., 2006; Gordon et al., 2016; Soares, 2017; Gracia and Freeman, 2018) and ovariectomized rodents (de Chaves et al., 2009; Li et al., 2014; Schoenrock et al., 2016), in whom levels of key sex hormones are substantially lower, increased anxiety and depressive behaviors have been noted.

### Effects of Exogenous Estrogen Therapies on Mood

Estrogen-containing treatments have been shown to improve mood or attenuate depressive symptoms in humans (Schmidt et al., 2000; Soares et al., 2001; Poromaa and Segebladh, 2012; Maki et al., 2019) and to reverse at least some ovariectomy-induced pro-depressive changes in rodents (Bernardi et al., 1989; Galea et al., 2001; Walf and Frye, 2009; Schiller et al., 2013; Li et al., 2014; Hiroi et al., 2016), suggesting pro-resilience benefits. Estrogens, especially the most potent naturally circulating estrogen 17β-estradiol (E2), are known to induce dendritic spine plasticity and neuronal complexity, facilitate neurogenesis, regulate brain region volume and activity levels, and impact key neurotransmitter and growth factor systems implicated in depression, to name just a few examples (Galea et al., 2001; Maki and Resnick, 2001; Brinton, 2009; Walf and Frye, 2009; Wharton et al., 2012; Marrocco and McEwen, 2016; Engler-Chiurazzi et al., 2017). Yet, not all studies report beneficial impacts of exogenously administered estrogens on mood. Several studies have noted increased depression among women taking hormonal contraceptives (Duke et al., 2007; Skovlund et al., 2016; de Wit et al., 2020) though collective findings generally suggest that contraception exerts minimal effects on mood (Robakis et al., 2019). The realization of neurobiological and behavioral effects of estrogen-containing treatments depends on a number of factors including, but not limited to, age of the organism, etiology and duration of hormone depletion, type of estrogen, treatment route of administration, treatment regimen, and functional domain targeted (Engler-Chiurazzi et al., 2017). Consideration of these factors is of key importance when assessing mood-impacting effects of this hormone.

## Evidence of Immune Impacts on the Development and Persistence of Depression

The immune system supports the body’s response against infection, injury, and disease. This complex network of intercommunicating, interactive cells and their secretory factors coordinates across multiple organs to mount a rapid and appropriate response to a threat to homeostasis through complex signaling cascades and activation/regulation sequences; the reader is directed to several excellent reviews that thoroughly describe the complexities of this system in detail (Chaplin, 2010; Marshall et al., 2018). Understanding of the complexity of neuroimmune mechanisms within the central nervous system (CNS) has grown rapidly in recent years. Although once considered “immune privileged”, a compelling body of literature indicates that the CNS and the peripheral immune systems engage in bidirectional communication, profoundly influencing one another during homeostasis and in pathological/diseased states (Lucas et al., 2006; Pavlov et al., 2018), including those associated with chronic stress and MDD (Dantzer, 2018). Microglial cells, the resident immune cells of the CNS, represent a particularly well-studied neuroimmune cascade mediator. Their actions as well as the contributions of other key CNS components (i.e., astrocytes, oligodendrocytes, perivascular macrophages, neurons, and endothelial cells) to the local neuroinflammatory cascade in response to CNS perturbation have been extensively described elsewhere (Ousman and Kubes, 2012; Ransohoff et al., 2015; Morimoto and Nakajima, 2019). Therefore, we will focus our discussion on the contributions of peripheral immune components to mood and MDD.

The peripheral innate immune response is characterized by rapid and non-specific activation of pattern/danger recognition receptors on innate immune cells to initiate phagocytosis of non-self antigens, secrete a variety of signaling factors including cytokines and chemokines, and/or function as antigen presenting cells to trigger adaptive immune activation (Chaplin, 2010; Marshall et al., 2018). Inflammation driven by innate immune system components, particularly macrophages, in modulating mood is now well established (Adzic et al., 2018). Chronic inflammation is implicated in a variety of mood disorders, leading to the emergence of the “macrophage/monokine theory of depression” (Dey and Hankey Giblin, 2018). For instance, depressive phenotypes have been consistently reported both among patients receiving proinflammatory cytokine treatment regimens and in preclinical models (Pryce and Fontana, 2017). As well, elevated levels of circulating cytokines, principally tumor necrosis factor (TNF)-α, interleukin (IL)-1β and IL-6, have been repeatedly reported among some subsets of depressed clinical populations (Dowlati et al., 2010; Köhler et al., 2017). Elevated levels of these inflammatory biomarkers are often associated with poor responsiveness to 5-HT targeting interventions (Arteaga-Henríquez et al., 2019), and anti-depressant treatment has been shown to reduce proinflammatory cytokine levels among treatment-responders or in preclinical models of immune challenge (Roumestan et al., 2007; Arteaga-Henríquez et al., 2019). Finally, compared to placebo, antidepressant treatment with co-administration of agents of anti-inflammatory action, such as non-steroidal anti-inflammatory drugs, statins, or cytokine inhibitors, improved depressive symptoms and MDD remission rates (Köhler-Forsberg et al., 2019). Mood benefits among depressed patients were even realized when these anti-inflammatory agents were administered as monotherapies (Köhler-Forsberg et al., 2019).

The complement system, an innate Immune arm that amplifies the recruitment signals initiated by other innate immune players, labels non-self antigens to facilitate immune-induced attack on these cells and mitigates the spread of the infection via membrane dysfunction-induced cell death (Rus et al., 2005), is also impacted by stress and depression. Indeed, levels of C3c and C4 complement as well as several other positive acute phase proteins including α1-antitrypsin and haptoglobin are elevated in depressed populations, while negative acute phase proteins like albumin are reduced (Kronfol and House, 1989; Maes et al., 1992b; Song et al., 1994).

The adaptive immune arm represents a delayed, antigen-specific response that targets intracellular infection/damage, amplifies and also resolves inflammatory cascade responses, and facilitates antigen memory (Chaplin, 2010; Marshall et al., 2018). Though evidence supporting a role for the peripheral adaptive immune system in modulating mood was slower to evolve due in part to the historical perception that lymphocytes are largely absent from brain parenchyma, T and B cells have also been implicated in response to CNS injury and disease, in the control of some normal brain functions and more recently, in MDD (Maes, 2011; Herkenham and Kigar, 2017; Dantzer, 2018). Indeed, many adaptive immune cells express the cellular machinery to respond to stimulation by the stress hormone, cortisol, and elevated cortisol levels, like those associated with a host of mood disorders, tend to be immunosuppressive (Gruver-Yates et al., 2014; Kovacs, 2014). Importantly, chronic stress is known to affect lymphocyte numbers/function in both humans suffering from mood disorders and in preclinical populations exposed to stressful conditions (Yin et al., 2000; Domínguez-Gerpe and Rey-Méndez, 2001; Frick et al., 2009; Scheinert et al., 2016). Lymphocytes are also profoundly impacted by 5-HT, at least in the periphery (Herr et al., 2017).

That peripherally derived T cells are now appreciated to be present in healthy brain parenchyma and can also infiltrate CNS tissue in response to injury or autoimmune disease has fostered major interest in the role of antigen-specific adaptive immunity in normal and abnormal brain function, including within the context of chronic stress and depression (Fletcher, 2010; Maes, 2011; Filiano et al., 2017; Herkenham and Kigar, 2017; Rayasam et al., 2018). Several seminal observations among depressed patient populations reported increased numbers of T helper/inducer cells and shifted ratios of CD4^+^/CD8^+^ T cells (Darko et al., 1988; Schleifer et al., 1989; Maes et al., 1990). Further, studies in lymphocyte-deficient mice (nude, scid or Rag^–/–^ mice) have noted deficits in adaptability to stress and reconstitution with lymphocyte populations generally implicated the absence of T cells in mediating these deficits in a subset-specific way (Cohen et al., 2006; Beurel et al., 2013; Rattazzi et al., 2013; Brachman et al., 2015; Clark et al., 2016). For example, (primarily) T lymphocytes from stress-exposed mice can modify the behavioral response to stress when adoptively transferred into lymphocyte deficient subjects (Brachman et al., 2015). T cells also robustly respond to glutamatergic signaling (Ganor and Levite, 2012), a neurotransmitter system that is emerging as a key contributor to MDD and a principle target for novel, fast acting antidepressants (Wang Y. T. et al., 2021).

The B cell component of the adaptive immune system may also play an important role in modulating both normal CNS function as well as the response to stress. Historically there were inconsistencies with regards to whether B cells were changed in depressed populations. However, methodological advances in measurement of these populations has revealed blood B cell number alterations in the context of mood disorders, including chronic academic stress, MDD, bipolar disorder, and panic disorder (Darko et al., 1988; Maes et al., 1992b; Schleifer et al., 2002; Robertson et al., 2005; Pavón et al., 2006; McGregor et al., 2016; Ahmetspahic et al., 2018). Further, some studies have reported B cell responsiveness among MDD patients given monoamine-modulating antidepressant interventions (Hernandez et al., 2010; Ahmetspahic et al., 2018). These observations have been successfully recapitulated in a recently published preclinical study leveraging the chronic social defeat stress model (Lynall et al., 2021). Indeed, pioneering work from the Clathworhy group (Lynall et al., 2021) reported that chronic stress increased splenic B cell activation and increased meningeal monocytes, while meningeal B cell counts were reduced. From a mechanistic perspective, like T cells, B cells have been shown to express 5-HT receptors and the 5-HT transporter, indicating that these cells may even take up this key MDD-associated neurotransmitter and transport it to distant sites (Meredith et al., 2005; Herr et al., 2017). Whether the brain is one of these is yet to be determined. As well, growth factors, such as brain derived neurotrophic factor, have been implicated in the manifestation of MDD (Yang et al., 2020), and their stimulation is critical for B cell development (Schuhmann et al., 2005; Fauchais et al., 2008). Given the crucial role of B cells in antigen presentation to T cells, their ability to facilitate T cell activation, and emerging understanding of their immunoregulatory impacts, additional exploration of their role in the response to stress is warranted.

Key functional activities of B cells, such as antibody secretion, may also be altered by stress in an antibody subclass-specific way (Kronfol and House, 1989; Joyce et al., 1992; Song et al., 1994; Gold et al., 2012). For example, relative to mentally healthy control subjects, Gold and colleagues (Gold et al., 2012) noted that depressed populations displayed reductions in serum IgA, but not IgM or IgG levels, while Joyce et al. (Joyce et al., 1992) reported increased IgA. Methodological differences between sample populations and measurement approaches may account for some of the discrepancy between these studies. The critical role of hypothalamic-pituitary-adrenal axis dysregulation and altered cortisol secretion in the manifestation of MDD is well established (Krishnan and Nestler, 2008). Physiological states associated with high levels of circulating cortisol, such as hypercortisolism (Sarcevic et al., 2020) or treatment of patients with corticosteroid-based interventions, shifts serum antibody profiles relative to healthy controls (Griggs et al., 1972; Settipane et al., 1978). As well, neuronal surface autoantibody expression has been implicated in a number of neuropsychiatric conditions, MDD included (Zong et al., 2017).

## Estrogenic Impacts on Immune Function During Distinct Reproductive Milestones Across the Female Lifespan

**FIGURE 1:**
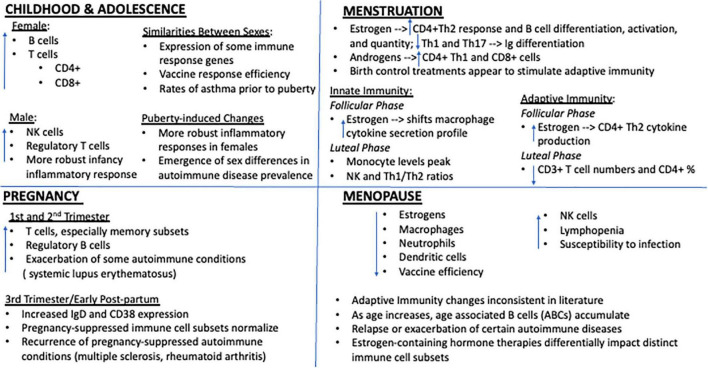
Key immune system impacts of estrogen at distinct female reproductive milestones. Immune function is profoundly impacted by genetic sex and variations in estrogen. Though a few subtle differences have been reported during childhood, prior to puberty onset immune cell counts are generally similar between males and females and any differences appear to have little functional impact on overall immunity. However, beginning with adolescence and the onset of menstruation, marked sex differences in immune cell ratios and response profiles emerge. Generally, females display a more robust inflammatory response to immune challenge, rendering them potentially more resilient to the negative consequences of infection but also more susceptible to certain autoimmune conditions. High concentrations of estrogen, whether they be due to natural shifts in circulating levels across the cycle or via administration of estrogen containing exogenous treatments, appear to exert cell-type specific effects with regards to key immune players, generally potentiating adaptive immunity. Falling estrogen levels with the transition to reproductive and immunosenescence also imparts profound consequences for immunity and is associated with dramatic shifts in peripheral immune cell profiles, autoimmune disease manifestation, and susceptibility to immune challenge.

Sex differences in immunity are well documented, and hormone influences, including those of estrogens, have been shown to impact immune function throughout adulthood (Pennell et al., 2012; Klein and Flanagan, 2016). Immunological impacts of genetic sex and of estrogenic stimulation across key reproductive milestones are described in the following sections and have been summarized in Figure 1.

### Mechanisms of Estrogen Regulation of Immunity

Estrogenic signaling is regulated by two nuclear estrogen receptors (ER), ERα and ERβ, both of which are expressed on a variety of immune cell types and tissues. For instance, ERα is widely expressed in bone marrow thymocytes and hematopoietic cells, while ERβ expression appears to be limited to the thymus, lymphocytes in lymph nodes, and the spleen in mid-gestational fetuses (Khan and Ansar Ahmed, 2015; Moulton, 2018; Rubinow, 2018; Zhang et al., 2020). Estrogens regulate immune cell number and function likely via an ER-dependent mechanism. When human lymphocytes were administered 17β-E2, CD45 and CD45RO isoform RNA expression were increased, an effect that was blocked with co-treatment of ER antagonists (Zhang et al., 2020). Less potent naturally circulating estrogens also appear to exert similar regulatory effects on immune cells. For example, estriol, at levels similar to the first trimester of pregnancy (2 ng/mL), increased levels of venous blood CD4^+^FoxP3^+^ T regulatory cells and decreased levels of CD4^+^RORC^+^ Th17 lymphocytes were seen in women of reproductive age (Shirshev et al., 2019).

### Sex Differences in Immunity During Early Life and Puberty

Some subtle sex differences in childhood immunity have been reported. For example, splenocyte response to cell surface-receptor-independent mitogenic combination of phorbol ester and ionomycin was greater in female mice at 3 weeks old, but was greater for 4-6 week old male mice (Rosen et al., 1999). Furthermore, a study on healthy Asian children noted that male babies showed 8% more natural killer (NK) cells at birth than females, while female newborns showed higher levels of CD3^+^ T cells (Lee et al., 1996). Between 1 and 6 years of age, girls had somewhat higher numbers of lymphocytes, B cells, and CD3^+^, CD4^+^, and CD8^+^ T cells, while boys had higher NK, activated T cells, and CD4^+^ T cell counts (Lee et al., 1996). In contrast, Lisse et al. found that West African boys show higher levels of CD8^+^ cells and lower CD4^+^/CD8^+^ ratios than girls (Lisse et al., 1997). Despite the discrepancy between these studies, prior to the onset of puberty, it is generally thought that the immune systems of male and female organisms exhibit few robust sex differences in immune cell counts or function (Robinson et al., 2014; Sharma et al., 2019). Indeed, splenic expression of some innate immune response genes was greater in pre-pubescent male mice, though the differences were not statistically significant and expression of adaptive immune response genes was generally similar between the sexes (Lamason et al., 2006). There also appear to be no sex differences in the vaccine response during childhood (Vom Steeg et al., 2019). This variability in the literature warrants future study to clarify the extent to which these observations replicate across study populations and translate to impact immunity overall during childhood.

The pubertal transition to reproductive capacity and the associated dramatic increases in sex hormone levels marks a period of substantial change in the immune system, changes that may exert functionally significant effects with regard to immune function in developing children. For instance, studies have noted increased numbers of circulating NK cells, CD4^+^ T cells, and B cells among girls, but higher CD8^+^ T cell numbers among adolescent boys as well as distinct response profiles of cultured peripheral blood mononuclear cells derived from male vs. female donors to phytohaemagglutinin stimulation (Lee et al., 1996; Uppal et al., 2003; Abdullah et al., 2012). There are numerous functional consequences of these puberty-induced sex differences in response to antigen challenge. Inflammatory responses to infection or toll-like receptor stimulation appear to be stronger in females than in males (Seillet et al., 2012; Robinson et al., 2014), with females showing increased gene expression of interferon-gamma, lymphotoxin beta granzyme A, IL-12 receptor beta2, and granulysin (Hewagama et al., 2009). Similar findings have been found preclinically where, in post-pubertal mice, following stimulation with ovalbumin and anti-CD3/CD28 antibodies, IL-4, IL-5, IL-13 were all significantly higher in female bronchial lymph node cells than in male cells (Okuyama et al., 2013). Additionally, IL-5 production from stimulated CD4^+^ T cells was significantly increased in females compared to males. Viral challenge with the mimetic polyinosinic:polycytidylic acid induced greater sickness behavior in post-pubertal males than females (Sharma et al., 2019). However, changes in body temperature and central c-fos expression were more prevalent in female mice, and gonadectomy both worsened sickness behavior and altered temperature in both sexes. Efficiency of vaccination has also been tested in murine models with adult female mice having greater antibody response to the vaccination and an increased number of antigen-specific hepatic CD8^+^ T cells compared to young mice (Vom Steeg et al., 2019). Another functional consequence relates to the prevalence of immune-associated diseases, especially asthma. Indeed, despite having similar numbers during childhood, adult females exhibit a 6.2% prevalence of asthma while males exhibit a 4.3% prevalence (Vink et al., 2010). Evidence supports that asthma responses and estrogen are largely correlated (Melgert et al., 2007) and that estrogen contributes to the innate macrophage polarization, thus leading to greater allergy response (Keselman et al., 2017).

### Immune Variation Across the Ovulatory Cycle of Reproductively Capable Organisms

Innate immune cell number and function display a complex pattern throughout the menstrual cycle. For example, peripheral levels of NK cells along with their cytotoxic potential became heightened during the luteal phase, when estrogen levels begin to decline but progesterone levels tend to be high (Lee et al., 2010). Monocyte numbers also appear to peak during the luteal phase while circulating neutrophil levels decline during menstruation (Pennell et al., 2012). Overall, estrogen seems to enhance, while progesterone and androgens tend to suppress proinflammatory innate immune responses (Roberts et al., 2001; Musabak et al., 2003; Arruvito et al., 2008; Shepherd et al., 2020).

Adaptive immunity also displays dynamic changes across the menstrual cycle. Estrogens generally have stimulatory effects on lymphocyte presence, concentration, and function (Lee et al., 2010; Oertelt-Prigione, 2012; Pennell et al., 2012; Rodriguez-Garcia et al., 2013; Moulton, 2018) though cell type and tissue-specific effects of estrogen stimulation have also been suggested (Pung et al., 1985; Chen et al., 2015). For instance, increased levels of estrogen are thought to stimulate overall CD4^+^ Th2 cytokine production in females (Ackerman, 2006; Pennell et al., 2012; Shah, 2012). Peripheral regulatory T cell counts were shown to be higher during the follicular phase when estrogen levels are typically highest (Moulton, 2018). Wegienka et al. (2011) noted that blood levels of the less potent naturally circulating estrogen, estrone, were positively correlated with regulatory T cell counts in asthmatic women. Peripheral blood CD3^+^ and CD4^+^ T cell percentages decrease in the luteal phase, when estrogen levels are low relative to those of progesterone (Lee et al., 2010). Inhibitory effects of estrogens have also been noted within certain immune cell subtypes. Indeed, estrogen exposure inhibits Th1 cytokine proliferation and Th17 differentiation (Chen et al., 2015).

Though information regarding B cell changes across the menstrual cycle is more limited, converging evidence suggests that estrogen stimulates B cell differentiation and activation, increases B cell numbers, and enhances their function (Verthelyi, 2001; Oertelt-Prigione, 2012; Moulton, 2018). For example, B cell activation by 17β-E2 generally induces higher levels of Ig synthesis (Franklin and Kutteh, 1999; Pennell et al., 2012) specifically in B cells found in bone marrow and the spleen (Moulton, 2018). In mice treated with sustained slow-release 17β-E2-containing silastic implants (4-6 mg) resulting in levels comparable to those achieved during murine pregnancy, numbers of antibody-secreting plasma cell numbers increased dramatically, and secretion of various immunoglobulins and autoantibodies increased (Verthelyi and Ahmed, 1998). This estrogen-driven B cell hyperactivity may contribute to the development of autoimmune diseases (Pennell et al., 2012).

Sex hormone type also appears to influence female immunity in a cell subtype-specific way. Indeed, while it is widely accepted that estrogens usually correspond with an increased CD4^+^ Th2 cell response, androgens promote CD4^+^ Th1 and CD8^+^ cell responses (Ackerman, 2006; Pennell et al., 2012; Guven Yorgun and Ozakbas, 2019). As for progesterone, increased levels during the luteal phase sometimes correspond to increased NK cell levels, unchanged Th1/Th2 ratios, decreased CD3^+^ and CD4^+^ T cell percentages, and increased serum levels of the anti-inflammatory IL-1 receptor antagonist (Lee et al., 2010; Vetrano et al., 2020). Despite this, in one study, serum CD4^+^/IL10^+^ regulatory T cells displayed heightened responses when progesterone levels were elevated in the late follicular and luteal phases (Weinberg et al., 2011). These findings reveal the significance of distinguishing between the different immune cell subtypes in how they react to steroid hormone stimulation in distinct target tissues and in response to various immunological challenges.

### Pregnancy-Associated Impacts to the Immune System

Tight regulation of the maternal immune response are key contributors to pregnancy success; historically, immune responses during pregnancy were thought to be suppressed to allow for a semi-allogeneic fetus (Racicot et al., 2014). However, this previously held notion has been reevaluated as additional findings implicating sex hormone regulation of immune responses have emerged in recent years (Mor and Cardenas, 2010). Indeed, immune contributions to the development of the decidua and placenta and the maintenance of the maternal-fetal interface is required for a successful pregnancy (Hsu and Nanan, 2014). Nair et al. (2017), and there is growing appreciation that dynamic shifts in maternal sex hormone levels may, at least in part, contribute to observed shifts in gestational immunity (Robinson and Klein, 2012). Uterine immune cells, including NK cells, macrophages, T cells, dendritic cells, mast cells, and B cells, are necessary for the normal formation of placenta beds and appear to play a key role in converting high-resistance, low-flow vessels to low-resistance, high-flowing vessels in spiral arteries in the placental bed (Faas and De Vos, 2018). Maternal monocytes and macrophages obtain a unique phenotype throughout pregnancy that allows them to retain immunological tolerance and permit hormone–immune cell interactions, both of which are required for progression of the fetus inside the uterus (Mendoza-Cabrera et al., 2020).

It is thought that the increase in steroid hormone levels throughout pregnancy modulates inflammatory responses at the maternal fetal interface, and E2, estriol, and progesterone influence the transcriptional signaling of those responses (Robinson and Klein, 2012). During the first trimester, levels of placental-derived estrogen increase sharply and contribute significantly to the development of organs and other bodily systems in the fetus. T cell subsets are profoundly affected by these changes. Early in pregnancy, the increase of regulatory T cells supports the development of a semi-allogeneic fetus protected from maternal immune rejection by restraining inflammation during the shift from proinflammatory to anti-inflammatory immunity (Krop et al., 2020). CD25^+^/CD4^+^ T regulatory cell numbers reach a peak during the second trimester, and it is thought that these cells allow the maternal immune system to respond to the developing fetal organs within the uterus (Somerset et al., 2004; Lima et al., 2017). Further, appropriately titrated T cell populations early in pregnancy may contribute to fetal viability. Indeed, when Lissauer and colleagues (Lissauer et al., 2014) evaluated circulating T cell subsets across distinct pregnancy stages, they observed that about 60% of Th17 cells in the body during pregnancy were found during the first trimester of pregnancy, though no changes in Th1 or Th2 T cell subsets were noted across the gestational and postpartum period. Th1 and Th17 cells numbers were elevated among women with recurrent miscarriage, suggesting that these cell types may serve as important targets to improve gestational success (Lissauer et al., 2014). Memory T cells are also increased during the first trimester and promote fetal-maternal tolerance (Kieffer et al., 2019). It is thought that insufficient numbers of memory CD4^+^ T cells contribute to pregnancy complications such as preeclampsia, gestational diabetes, and premature labor (Lim et al., 2019). Following pregnancy, it can take three to four months for cells to return to normal function after delivery, and inhibition of helper T cells and NK cells appears to last for the first few months.

Like T cells, B cells also support the semi-allogeneic fetus while protecting the mother and fetus against infection (Muzzio et al., 2013). Regulatory B cell numbers similarly increase during the first trimester, limiting proinflammatory responses (Esteve-Solé et al., 2018). B cell activation factors, which facilitate the inflammatory response, are also increased, likely in support immune tolerance of the semi-allogeneic fetus (Wang L. et al., 2021). B cells also appear to impact immunity during later stages of pregnancy and following parturition. Lima and colleagues examined healthy pregnancies and determined the degree of activation of different B cell subsets, reporting increases in CD38^+^ and IgD markers of B cell activation during the third trimester of pregnancy and postpartum period (Lima et al., 2016).

### Immune Shifts During Female Reproductive Senescence and Aging

Aging plays a significant role in modulating the function of the immune system and is associated with deterioration of immunity seen in the elderly (Fulop et al., 2017). Immunosenescence cascades have been reviewed elsewhere (Xu et al., 2020) but in brief, key immunosenescence characteristics include inflammaging, lymphopenia, higher susceptibility to infection and poor vaccine response (Ghosh et al., 2014). Within the adaptive immune system, aging is associated with an increase in differentiated memory T cells, effector T cells, senescent CD8^+^CD28^–^ T cells, and age-associated innate-like B cells, but a decrease in most B cell subsets and the ratio of CD4:CD8 T cells, to name just a few examples (Weyand and Goronzy, 2016). Collectively, these and other senescence-related changes diminish the ability of the immune system to protect against certain infections and cancers and may accelerate the development of certain diseases, rendering older populations at-risk for a host of immunological challenges. It was historically presumed that this senescence-associated shift in immunity occurred at a similar rate and manner, regardless of sex (Aiello et al., 2019). However, rising life expectancies have revealed that men and women experience these consequences along different trajectories; emerging evidence suggests sex-specific and potentially profound consequences of immunosenescence (Gubbels Bupp et al., 2018; Márquez et al., 2020). Indeed, it is now appreciated that female innate immune systems appear to age at a faster rate, whereas the adaptive immune systems of men age at a faster rate (Ghosh et al., 2014).

Age-related shifts in reproductive function likely influence the function of the immune system during aging, and the sex-specific nature of this transition period may account for differences in male and female immunosenescence. Indeed, while men experience andropause, a gradual reduction in circulating testosterone over the course of several decades (Kevorkian, 2007), women experience a more accelerated transition. Menopause marks a period of reproductive senescence in a woman’s life when the ovarian oocytes have become depleted, and sex hormones are no longer produced by the ovaries (Keppel and Wickens, 2004). As a result, the menstrual cycle becomes irregular and eventually terminates while levels of estrogens and progestins drastically decline. Natural menopause is a normal part of aging that typically occurs in the fourth and fifth decade of life and can take place over the course of only a few years. Still others undergo surgical menopause to remove the ovaries when there is an increased likelihood of cancer, infection or endometriosis (“Medical Causes of Menopause”), resulting in an accelerated reproductive senescence.

The distinct trajectory of female reproductive senescence has important impacts with regard to immune function during aging. In comparison to men of a similar age or to reproductively capable women, post-menopausal women are disproportionately affected by certain autoimmune disorders and have an increased susceptibility to infection with aging (Fairweather et al., 2008; Gubbels Bupp et al., 2018; Maglione et al., 2019). Estrogen deficiency has been implicated in many senescence-associated changes seen in the immune cells, such as the increase in proinflammatory markers IL-1, IL-6 and TNF-α (Gameiro et al., 2010), and low levels of estrogen are linked to higher levels of IL-17 produced by Th17 cells (Molnár et al., 2014). Following menopause, women undergo various changes in the levels of innate immune cells. Whereas the number of NK cells increases, their cytotoxic capacity is diminished (Albrecht et al., 1996; Ghosh et al., 2014; Toniolo et al., 2015). Further, the number of macrophages, neutrophils and dendritic cells decreases (Ghosh et al., 2014; Toniolo et al., 2015). Macrophages are vital, as they aid in the conversion of proinflammatory phenotypes to anti-inflammatory phenotypes, and estrogens help to prevent the effects of proinflammatory agents on the functions of macrophages by accelerating the resolution phase of inflammation in these cells (Toniolo et al., 2015; Villa et al., 2015). E2 also seems to decrease the rate of apoptosis in neutrophils as following menopause, neutrophils numbers have been shown to decrease as the rate of apoptosis increases (Chen et al., 2016).

Menopause is also associated with significant shifts in adaptive immunity. As reviewed in Gubbels Bupp et al. (2018), some studies report a decrease in total lymphocyte counts in postmenopausal women (Giglio et al., 1994; Kamada et al., 2000), while other studies have shown that numbers of some lymphocyte subsets are significantly higher in postmenopausal women (Chen et al., 2016; Abildgaard et al., 2020). Further, levels of functioning CD4^+^ T and B cells decrease, while numbers of exhausted and senescent cells rise, whether the etiology of menopause is surgical or transitional (Giglio et al., 1994; Gameiro et al., 2010; Gubbels Bupp et al., 2018; Maglione et al., 2019; Abildgaard et al., 2020; Vrachnis et al., 2021). However, it has also been shown that thirty days after surgical menopause via total abdominal hysterectomy and bilateral salpingo-oopherectomy, patients displayed increased levels of CD8^+^, but decreased levels of B cells and a reduced CD4^+^/CD8^+^ T cell ratio (Kumru et al., 2004). Other conflicting literature has noted a decrease in naïve CD8^+^ T cells, but an increase in memory or activated T cells in postmenopausal women compared to pre-menopausal women or women taking hormone replacement therapy (HRT) (Kamada et al., 2000; Engelmann et al., 2016; Vrachnis et al., 2021). In regards to the function of B cells, E2 enhances certain aspects of humoral immunity (Gameiro et al., 2010). Aged women tend to accumulate more innate-like age-associated B cells (ABCs) than young women and men of any age, and there is a relationship between ABCs, viral infections, autoimmunity and a proinflammatory state (Rubtsova et al., 2015; Gubbels Bupp et al., 2018). ABCs are known to originate from follicular B cells and show a bias in females, due to hormones and X chromosome-encoded genes, but the mechanisms that cause the production and accumulation of ABCs are still unknown and need to be further investigated (Rubtsova et al., 2015).

### Effects of Exogenous Estrogen-Containing Treatments on Immune Function

Immune cells not only respond to endogenously secreted estrogens; estrogen-containing contraceptives, commonly used for pregnancy prevention, hormonal imbalances, and menstrual cycle regulation, also impact immunity. For example, compared to untreated women, women taking the oral contraceptive pill, Ortho Novum 777 (containing ethinyl estradiol and norethindrone), had higher Ig levels, implicating these hormones in promoting B cell activity (Franklin and Kutteh, 1999). In another small study evaluating respiratory performances of thirteen asthmatic women, blood regulatory T cell counts were higher among contraceptive treated women, and this was associated with less intense asthmatic symptoms (Wegienka et al., 2011; Vélez-Ortega et al., 2013). Estrogen-containing contraceptives administered vaginally also impact the local immune environment. Indeed, Hughes et al. noted that the NuvaRing^®^ (0.12 mg etonogestrel/0.015 mg E2 per day) was associated with increased T cell- related proteins, granulysin and granzyme B in cervicovaginal fluid, indicating that, similar to during phases of heightened estrogen in the menstrual cycle, estrogen has a stimulatory effect on vaginal T cell response when locally administered. Yet in mice, when the synthetic estrogen, diethylstilbestrol, was administered subcutaneously for five consecutive days, T cell proliferation and IL-2 production in the spleen both declined (Pung et al., 1985), implicating species or estrogen subtype differences with regards to exogenous estrogen impacts to immunity.

Though menopausal HRT is commonly prescribed to attenuate the negative vasomotor and vaginal symptoms of menopause, it may also be a potential therapeutic option to modify menopause-related shifts in immune system function (Ghosh et al., 2014); the complexities associated with HRT impacts to the brain and immunity have been extensively reviewed elsewhere (Abdi et al., 2016). As an example, postmenopausal women taking estrogen and progestin-containing HRT have been reported to have higher numbers of lymphocytes and B cells specifically, but maintain low levels of CD4^+^ T cells, and exhibit a decrease in CD8^+^ cells resulting in an increase in the ratio of CD4^+^/CD8^+^ T cells; naïve and memory/activated T cell numbers generally remained consistent (Kamada et al., 2000; Yang et al., 2000; Porter et al., 2001; Kumru et al., 2004). These HRT-induced immune cell impacts may be effective in alleviating the symptoms associated with menopause or autoimmune disease, as well as the risk for developing certain disorders, especially when used within 10 years of experiencing symptoms if the woman is under 60 years old (Stopińska-Głuszak et al., 2006; Cagnacci and Venier, 2019). Taken together, these data indicate that exogenous estrogen treatment has significant immunological consequences, which may in turn impact women’s susceptibility to systemic infection or autoimmune disease (see below); additional investigation in this research domain is clearly warranted.

## Sex, Estrogen and Autoimmunity – Consequences of Estrogenic Impacts to the Female Immune System

Though the evidence described above reveals robust sex-specific differences in immune responses, may at times, provide some advantages to infection for female organisms, maladaptive consequences have also been indicated. Indeed, women shoulder a disproportionate burden of some autoimmune diseases and several reviews extensively explore the topic of sex differences in the prevalence of autoimmunity (Lateef and Petri, 2012; Pennell et al., 2012; Ortona et al., 2016; Moulton, 2018; Keestra et al., 2021). For instance, with a typical age of disease onset occurring during puberty, the prevalence of systemic lupus erythematosus (SLE), an autoimmune condition associated with widespread inflammation, in prepubertal girls is only double that of boys; by adulthood, the ratio of female to male patients has increased to 9:1 (Ngo et al., 2014; Moulton, 2018). Further, SLE-associated flare-ups during pregnancy are common (Petri, 2020). That estrogen stimulation promotes a Th2 immune phenotype may further contribute to the increased prevalence of Th2-mediated autoimmune diseases such as SLE (Ackerman, 2006). For instance, regulatory CD4^+^ T cells from female SLE patients showed reduced FoxP3 expression when incubated with physiological levels of E2, suggesting that high E2 levels may place women at an increased risk due to the presence of fewer immune regulatory cells (Singh and Bischoff, 2021). Among SLE patients, HRT has been found to increase the amount of mild, but not severe, flares (Lateef and Petri, 2012).

A sex-specific burden of multiple sclerosis (MS), a chronic, progressive, demyelinating inflammatory autoimmune disease associated with a myriad of degenerative sensori/locomotor and cognitive deficits, has also been documented (Goldenberg, 2012). Indeed, a woman’s risk for developing MS increases after the pubertal transition, an effect linked to increasing levels of estrogens given that MS symptomology appears to decrease in intensity during the luteal phase of the menstrual cycle, when estrogen levels are low (Moulton, 2018; Keestra et al., 2021). Additional clarity regarding the contributions of sex hormones alone and in combination is warranted as perplexingly, some studies note greater MS symptomology and worsened cognitive function in the premenstrual phase when sex hormones are generally at their lowest levels (Guven Yorgun and Ozakbas, 2019; Keestra et al., 2021). As well, though relapse rates increase significantly by three months post-partum, pregnancy is typically associated with symptom remission (Confavreux et al., 1998). Short-term corticosteroid treatment to manage MS symptoms during late pregnancy is considered safe with regards to fetal outcomes such as risk of pre-term birth and low birth weight (Ramo-Tello et al., 2021). Whether this treatment impacts affective outcomes in the pregnant or post-partum mother is not clear and represents an important area of investigation, given that corticosteroid treatments are known to induce psychiatric symptoms such as mania, depression, psychosis, and cognitive changes (Brown and Chandler, 2001).

Rheumatoid arthritis (RA) is an autoimmune disease characterized by joint pain, painful swelling, fatigue and fever due to the immune system attacking its own healthy tissue (Bullock et al., 2018). RA is both more common and may be more severe in women than men (Walker, 2011; Pennell et al., 2012). Like MS patients, women with RA experience symptom remission during pregnancy but these effects are short-lived as women often experience disease aggravation following parturition (Ostensen et al., 1983). The typical age of RA onset in women is during the menopausal transition, and an early age at menopause is associated with an increased likelihood of RA (Goemaere et al., 1990; Desai and Brinton, 2019). This observation may be attributed to the loss of endogenous estrogen that women experience during menopause. Menopausal RA patients taking HRT do not appear to display increased flare-ups and may even experience improved disease symptomology (Holroyd and Edwards, 2009). Similar effects have been demonstrated among pre-menopausal women, where oral contraceptive use did not prevent emergence of new disease but did reduce transformation of cases from mild to severe, suggesting beneficial effects of exogenously-administered estrogen-containing therapies against disease progression.

## Convergence of Sex, Estrogen and Immunity in Stress and Depression

The complexities of how biological sex or sex hormones and peripheral immunity converge to impact mood are beginning to be revealed. Sex-specific affective responses to peripheral inflammatory challenge generally suggest that female organisms may respond more robustly to immune activation (Bekhbat and Neigh, 2018). Indeed, inflammatory challenge with lipopolysaccharide (LPS; bacterial infection mimic) was associated with mood disruptions in women but not men (Moieni et al., 2015) and intranasal LPS administration induced depressive-like behavior and elevated hippocampal proinflammatory cytokine expression only in female rodents (Tonelli et al., 2008). However, this effect has not consistently been observed. For instance, following LPS challenge, while women displayed greater increases in proinflammatory IL-6 and TNF-α levels than men and men displayed higher levels of the typically anti-inflammatory cytokine IL-10, surprisingly affective consequences were similar among both sex groups (Engler et al., 2016). Similar observations were noted in preclinical studies where male and female rodents displayed similar depressive-like behavioral phenotypes despite robust sex-distinct effects on inflammatory and growth factor cascades in response to peripheral immune stimulation (Adzic et al., 2015; Brkic et al., 2017). Still, other reports suggest that males may be more susceptible to affective impacts of peripheral immune activation. Indeed, in male mice exposed to a LPS challenge, depressive-like behavioral changes along with altered brain proinflammatory cytokine mRNA levels were observed at 24 h, and hippocampal apoptosis was shown at 28 days later, effects not observed in female mice (Millett et al., 2019; Rossetti et al., 2019).

Sex differences in peripheral circulating cytokine levels among clinically depressed populations or in preclinical models have also been reported. For instance, higher levels of C-reactive protein were associated with an increased risk of depressive transformation, and increased psychopathology among depressed women was associated with elevated levels of C-reactive protein where no such association was noted in depressed men (Köhler-Forsberg et al., 2017; Kim et al., 2021; Zainal and Newman, 2021). Genetic predispositions related to the immune system also appear to induce sex-specific risk factors for development of a depressive phenotype as IL-18 haplotype in women, but not men, is associated with increased threat-induced central amygdala reactivity (Swartz et al., 2017). Other cytokines that are associated with depressive phenotypes in females, or the responsiveness of depressed patients to antidepressant treatment, include IL-1β, and IL-6 (Carboni et al., 2019; Kim et al., 2021; Zainal and Newman, 2021). However, some inconsistencies regarding sex-specific differences in peripheral inflammation among depressed populations have been reported. For example, while Piantella and colleagues (Piantella et al., 2021) agreed with other literature that IL-6 was associated with higher depressive symptoms in women exposed to workplace stress, they observed that higher C-reactive protein levels were associated with depression only in men. Further, in a study of more than 1,800 patient samples, C-reactive protein was associated with MDD state only in men (Ramsey et al., 2016). The experimental heterogeneity associated with the study population and sample size, the stressor nature and severity being evaluated, the approach to measure cytokine levels, the post-stress measurement timeframe, etc., among evaluations reported in the literature indicate that additional work is needed to discern the utility of sex-specific cytokine biomarkers for depression.

Taken together, it appears that immune activation cascades in response to psychosocial stress differ between males and females, though whether the consequences of these distinct trajectories reliably manifest in differential mood-related disruptions between the sexes is not altogether clear. Further clarification of the parameters in which sex-specific mood impacts may be realized in the context of antigen-driven or sterile immune challenges is needed.

## Discussion: Challenges in Exploring Neuro-Immuno-Endocrine Interactions in the Context of Mood

**FIGURE 2:**
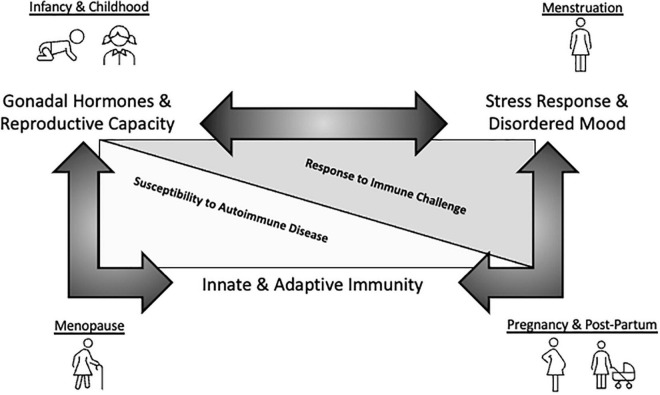
Schematic representation of mood-immune convergence across the female reproductive lifespan. A substantial amount of research has been dedicated to exploring how endocrine and immune factors impact mood separately. For instance, neuroprotective effects of estrogen in regards to MDD are well-established. Further, inflammatory insult and immune dysfunction are emerging as key contributors to disordered mood. Finally, genetic sex and estrogen clearly modulate immune system components, having important functional consequences for immunity across the reproductive lifespan. However, insight regarding how these two systems converge to impact mental health, especially during aging, is currently limited. This knowledge gap may be driven by experimental challenges associated with exploring these complicated interactions including, but not limited to, heterogeneity associated with the study population and sample size, the species used, the stressor nature and severity being evaluated, the approach to measure cytokine levels, the post-stress measurement timeframe, to name a few examples. Whether estrogenic influences on inflammatory activation cascades in the context of ‘sterile’ psychosocial stress-induced immune challenges result in sex-specific susceptibility to MDD during key reproductive milestones remains to be further interrogated and represents an exciting area of study.

As summarized above, genetic sex, estrogen, and the immune system significantly contribute to mood and mood disorders both individually and as converging, interactive factors (Figure 2). As this exciting field further develops, consideration of a number of limitations and challenges to probing these complex interactions in the context of mental health is warranted.

### Consideration of Relevant Biological Variables

First, though historical representation of both sexes in biomedical research has been lacking, there is increasing awareness among researchers regarding the need to consider sex as a biological variable and moreover consider how biological phenomena change as reproductive capacity shifts across the lifespan (Arnegard et al., 2020). Indeed, in 2015, the NIH (2015). announced requirements for the appropriate consideration of sex as a biological variable, incorporating this as a review criteria for all proposals submitted shortly thereafter. This policy change included requirements for the use of both sexes within study populations unless strong justification is provided as to why research questions being assessed could only be evaluated in one sex (e.g., exploration of ovarian function would preclude the use of only female organisms) as well as disaggregation of data analyses to observe sex-related trends and accurate reporting of data based on sex As part of NIH’s larger initiative to improve experimental rigor and reproducibility (Price and Duman, 2020), due consideration of other relevant biological variables is now also strongly advised (Lauer, 2016).

At present, the majority of research addressing the convergence of immune cells and sex or sex hormones on mood outcomes does not regularly factor in cyclicity stage, parturition experience, nor circulating levels of steroid hormones. As well, even when females are included in experimental designs, the majority of work in this area is conducted in young adult subjects prior to initiation of age-related immunosenescence cascades, potentially limiting translatability of the findings to older cohorts. While due consideration of key biological variables is not without its methodological challenges, there exists numerous aging or sex-based research centers of excellence around the United States (e.g., Nathan Shock Centers of Excellence in the Basic Biology of Aging, Tulane Center for Excellence In Sex-Based Biology and Medicine, and several workshops (e.g., International Symposium on the Neurobiology and Neuroendocrinology of Aging) providing training in the conduct of aging and/or sex-based research have been developed in recent years. In addition to informal laboratory based training, several publications laying out strategies are readily available (Bale and Epperson, 2017; Joel and McCarthy, 2017; Clayton, 2018). Especially given the profound age-related shifts in immune function, there exist numerous opportunities for productive research collaborations between immunologists, neuroendocrinologists, and biostatisticians to thoroughly address the convergence of sex, sex hormones, age, immune function, and stress responses. Continued progress is still needed (Woitowich and Woodruff, 2019; Arnegard et al., 2020), and additional incentivization of research specifically aimed at systematically addressing sex differences and the influence of sex hormones within the scope of mental health research will likely benefit the field.

### Complexities of Evaluating Mood and Modeling Human Mental Health Disorders Preclinically

Another significant challenge facing this area of study is that effectively modeling complex mood disorders such as MDD in a rodent is difficult (Krishnan and Nestler, 2008; Nestler and Hyman, 2010; Wang et al., 2017). Whereas the etiology of MDD can be varied in humans, ‘depressive-like’ states in rodents are typically experimentally induced via environmental, experiential, genetic, pharmacological, physical, social, or surgical manipulations. Many of the classic induction approaches, developed at the height of the monoamine hypothesis of depression, were aimed at revealing antidepressant efficacy novel drugs (Nestler and Hyman, 2010; Wang et al., 2017). Unfortunately, no single stress-induction approach fully recapitulates the heterogeneity of disease susceptibility and no one readout fully captures the behavioral and neurobiological pathology seen in human populations, though some newer paradigms have been developed that display better translational validity. For instance, the long-leveraged forced swim stressor results in near ubiquitous floating behavior, thought to be an indicator of behavioral despair/learned helplessness, while the chronic social defeat paradigm can effectively discriminate susceptible from resilient populations (Golden et al., 2011; Bogdanova et al., 2013).

As well, MDD is a psychiatric disorder associated with a variety of phenotypes, and many symptoms experienced by human patients (i.e., sadness, guilt, suicide ideation) cannot be directly evaluated in rodents. When MDD symptomology can be more effectively recapitulated preclinically (anhedonia, behavioral despair), the available murine tests of depressive-like behavior generally only probe one dimension of this heterogeneity. Evaluating depression phenotypes through the assessment of other impacted functions, such as cognitive domains, may provide additional insights (Hales et al., 2014; Price and Duman, 2020). Further, in contrast with the delayed response of antidepressants prescribed to patients in the clinic, acute treatment of mice with antidepressants is sufficient to alleviate depressive-like behavior in some of the commonly employed preclinical tests, though again some paradigms show response timing profiles similar to those observed in clinical populations (Golden et al., 2011; Willner, 2017).

Finally, methods used to induce stress phenotypes in rodents may confound readouts. For instance, a common behavioral readout of the chronic variable stress paradigm is sucrose preference, a measure of anhedonia, the results of which may be profoundly impacted by metabolic changes associated with brief food restriction, a commonly leveraged component of that stress-induction approach (Willner, 2017). As such, no single preclinical stressor paradigm or test for ‘depression’ fully recapitulates the complexity of the MDD phenotype nor the response profile to typically prescribed treatments given to alleviate symptomology.

Selection of the method to induce stress as well as the approach to determine behavioral, physiological, and neurobiological responses require careful consideration of the research question being posed. In alignment with recent guidance from the NIMH (2019) and in pursuit of current Research Domain Criteria (RDoC) recommendations (Maes, 2011), it is advisable to leverage tests where the underlying neurobiological circuitry is well understood rather than on the basis of “presumed congruence to human symptoms of mental illness”. Homological validity, that is capturing behavioral readouts that are species-relevant, should also be prioritized. The use of a behavioral battery of readouts within affective domains rather than a single assessment is also highly recommended to accurately capture the breadth and depth of a phenomenon, though test order should be an important consideration in their deployment (Powell et al., 2012). Composite behavioral battery z-scores should also be leveraged to capture overall impacts of stress on an organism as there is often substantial individual performance variability on unique readouts, especially among controls (Johnson et al., 2021). Behavioral readouts are best coupled with physiological readouts of stress, such as circulating corticosterone levels, metabolic alterations (such as attenuated weight gain), reduced self-care and health metrics, and shifts in circadian activity. There are also logistical complexities in preclinically modeling depression specifically in female organisms, including whether to consider stage of estrous cycle and reproductive capacity. Rigorous research portends the quantification of vaginal lavage to determine cyclicity and inclusion of Cycle Stage as an additional factor in statistical assessment of stress responses. Importantly, Johnson et al. (2021) did not identify a predicitive relationship between cycle stage and the stress response of their experimental and control animals. Further, though ‘non-brain’ measures showed more female-associated variability, a recent metanalysis of 311 articles did not report large-scale sex differences in neuroscience outcome measures and failed to identify increased variability in female rodents due to estrous cycle (Becker et al., 2016), suggesting that the impact of cycle stage on stress/neuroscience readouts may be relatively small. It is also important to consider that some stress paradigms are extremely difficult to apply to females and may need significant modification to be applied to appropriately. For example, chromogenic activation of the ventromedial hypothalamus was required to induce male aggressors to attack female test mice (Takahashi et al., 2017). When addressing age interactions in response to stress within females, careful consideration of species differences in the trajectory of reproductive senescence as well as the biological consequences of surgical hormone depletion is also warrented (Engler-Chiurazzi et al., 2017). Of note, there are potentially independent cognitive contributions of ovaries versus uterus (Koebele et al., 2019) and consideration of the entire reproducitve system is necessary to comprehensively discern immune-sex hormone interactions with mood. Finally, investigators should not limit themselves to studying only populations that display maladaptive stress response profiles but should also consider exploration of subjects that display stress resiliency; important understanding in this domain is actively being advanced (Russo et al., 2012; Faye et al., 2018).

### Sick as a Mouse: Can Rodent Models Effectively Recapitulate Human Immunity?

From an immunological perspective, consideration of the limitations of the experimental model leveraged to study the convergence of endocrine-immune factors within mental health is of paramount importance. First, species differences among humans vs. rodents in the development, total numbers, and functional ability of a variety of immune cell subsets have been long established and comprehensively discussed (Mestas and Hughes, 2004). For example, notable differences in innate immune responses including neutrophil defensin expression, toll-like receptor distribution, and macrophage function as it relates to nitric oxide, and natural killer cell inhibitor receptors for major histocompatibility complex I molecules between humans and rodents have been observed (Mestas and Hughes, 2004). Peripheral leukocyte profiles also vary by species such that up to 70% of immune cells in human blood are granulocytes (such as neutrophils) while lymphocytes make up approximately 30% of cells; monocytes are up to 10%, and other cell populations are more rare (Mestas and Hughes, 2004; Olin et al., 2018). In contrast, rodents display some sex differences in total blood leukocyte counts though importantly in both sexes lymphocytes, at between ∼75-90% for males and females, respectively, were the dominant immune cell type in circulation while neutrophil counts ranged between 24 and 8% (Doeing et al., 2003). Species differences in adaptive immune responses have also been reported, including variations in Fc receptor and Ig isotype expression, the regulation of T and B cell development, and the functional response of lymphocytes to antigen challenge (Mestas and Hughes, 2004). The consequences of these variations may be significant when attempting to address consequences of immunogens that exhibit host-specific patterns of infection, such as cytomegalovirus (Mestas and Hughes, 2004; Masopust et al., 2017), leading some researchers to suggest that rodents poorly recapitulate human injury or disease-associated inflammatory cascades and to advocate caution in the utilization of rodent models for immune-focused research questions (Seok et al., 2013).

As well, research mice are raised in specific pathogen free vivarium facilities that abide guidelines for cleanliness from regulatory organizations such as the United States Department of Agriculture and Association for the Assessment and Accreditation of Laboratory Animal Care accrediting bodies. While such practices support the health and welfare of laboratory rodents and promote reproducibility of data generated in a variety of fields, it is now recognized that pathogen free mice have immature immune systems that are functionally distinct from laboratory mice deliberately exposed to pathogens, from wild caught or pet-store reared mice, and from the human populations they are meant to model (Abolins et al., 2017; Masopust et al., 2017; Tao and Reese, 2017). For instance, adult humans have differentiated memory CD8^+^ T cell subsets that are not observed in laboratory mice raised in typical pathogen-free conditions; co-housing mice with more antigen experienced pet-store mice can “humanize” their immune profiles, potentially improving their translational validity (Beura et al., 2016). Importantly, antigen exposure history shapes the function of the immune system (Beura et al., 2016; Tao and Reese, 2017), an important consideration given that emerging evidence implicates a higher infection burden with several negative neurological and cognitive consequences across the aging trajectory. Whether these factors manifest in functionally significant impacts for immunity as it relates to mental health is not yet clear and will be an important area of future study as the field evolves. Increased interest among both scientists and funding organizations in the rethinking of the research pipeline, the utilization of “dirty” mice, and the deploying of novel sequencing methods that capture the complexity of immune responses to explore key research questions may reveal more translational insights in the coming years (Shultz, 2016; Tao and Reese, 2017; Wagar et al., 2018).

### Challenges Investigating Mood and Immunity in Human Populations

Many factors contribute to challenges in successful translation of preclinical findings to human populations. Here we will highlight variability in human immune profiles (Brodin and Davis, 2017) as well as mood disorder manifestations (Altemus et al., 2014). Immune profiles in middle aged adults evaluated longitudinally over the course of one year display some intra-individual variability that varies in magnitude from subject to subject and may be predictive of overall health (Lakshmikanth et al., 2020). Immune variability is also prevalent across individuals as immune profiles of the very young (Olin et al., 2018) and the very old (Kaczorowski et al., 2017) exhibit more heterogenous composition than do those of adults. As immune composition of monozygotic twins become increasingly distinct with time, the shaping of individual immune profiles is likely due to a combination of heritable and environmental influences (Brodin et al., 2015). Further, the numerous and sometimes vague or opposing diagnostic criteria used to identify clinically depressed patients leads to a potentially highly variable subject pool that likely reflects distinct MDD sub-phenotypes (Zimmerman et al., 2015; LeGates et al., 2019). To account for this variability when evaluating variables of interest, a straightforward statistical solution is to increase sample size (Keppel and Wickens, 2004). However, many of the seminal papers exploring immune profile variations among depressed and mentally healthy populations had stressed/depressed participant numbers of less than 50 (Darko et al., 1988; Maes et al., 1990, 1992a; Petitto et al., 1993; McGregor et al., 2016; Ahmetspahic et al., 2018). This potential under-sampling not only presents challenges to replicability of the significant differences of immune readouts revealed in each study, but could also indicate a lack of statistical power to detect more subtle differences (Keppel and Wickens, 2004). However, many studies do not report observed power nor effect sizes, limiting the ability to make such determinations. These collective factors may contribute to potentially large intra-individual differences that make evaluation of the convergence of mood and immune function a significant logistical challenge. Robust assessment of immune-mood-sex interactions with statistically powerful meta-analysis approaches will become more feasible as additional investigations are conducted.

## Conclusion

In summary, collective evidence addressing the unique affective contributions of genetic sex, sex hormones, reproductive capacity, and immunity has already expanded the prevailing ‘monoamine theory of depression’ and yielded improved understanding of the mechanisms driving disordered mood. Given the complex interactions that take place across the female lifespan between these systems, due consideration of how these factors acting in concert may converge to modulate mood is necessary. This will be made possible by adherence to new policies in the consideration of key biological variables, the inclusion of diverse subject populations and the reporting of findings based on population factors such as sex, reproductive experience, and age. The goal of this expanded appreciation for neuro-endo-immune factors in modulating mood is an increased appreciation for the mechanisms driving the manifestation of MDD and other mood disorders and revelation of novel, potentially sex or age-specific therapeutic interventions; we look forward to this outcome.

## Author Contributions

All authors listed have made a substantial, direct, and intellectual contribution to the work, and approved it for publication.

## Conflict of Interest

The authors declare that the research was conducted in the absence of any commercial or financial relationships that could be construed as a potential conflict of interest.

## Publisher’s Note

All claims expressed in this article are solely those of the authors and do not necessarily represent those of their affiliated organizations, or those of the publisher, the editors and the reviewers. Any product that may be evaluated in this article, or claim that may be made by its manufacturer, is not guaranteed or endorsed by the publisher.

## Abbreviations

5-HT: serotonin
E2: estradiol
ABC: age-associated B cell
CNS: central nervous system
ER: estrogen receptor
HRT: hormone replacement therapy
IL: interleukin
Ig: Immunoglobulin
LPS: lipopolysaccharide
MDD: major depressive disorder
NK: natural killer (cell)
rTMS: repetitive transcranial magnetic stimulation
SSRI: selective serotonin reuptake inhibitor
TNF: tumor necrosis factor.
